# ﻿A new Athysanini leafhopper genus from China (Hemiptera, Cicadellidae, Deltocephalinae), with description of two new species

**DOI:** 10.3897/zookeys.1260.173305

**Published:** 2025-11-19

**Authors:** Ke-Ting Duan, Mick D. Webb, Ji-Chun Xing

**Affiliations:** 1 Institute of Entomology, Guizhou University, Guiyang, 550025, China Guizhou University Guiyang China; 2 Guizhou Key Laboratory of Agricultural Biosecurity, Guiyang, 550025, China Guizhou Key Laboratory of Agricultural Biosecurity Guiyang China; 3 Department of Life Sciences, Natural History Museum, Cromwell Road, London SW7 5BD, UK Department of Life Sciences, Natural History Museum London United Kingdom

**Keywords:** Homoptera, identification key, leafhopper, morphology, new taxa, taxonomy

## Abstract

A new leafhopper genus of the tribe Athysanini (Hemiptera: Cicadellidae: Deltocephalinae), *Neonakaharanus***gen. nov.**, including two new species: *Neonakaharanus curvatus***sp. nov.** (type species) and *Neonakaharanus triangulatus***sp. nov.** are described and illustrated, and a key to species is provided. The type specimens of the new species are deposited in the Institute of Entomology, Guizhou University, Guiyang, China (GUGC).

## ﻿Introduction

The tribe Athysanini Van Duzee, 1892 is distributed across nearly all terrestrial ecosystems and is the most species-rich tribe of Deltocephalinae. At present, there are 214 valid genera and 1074 valid species distributed worldwide (Dmitriev et al. 2022). Due to the considerable size of this tribe and its morphological diversity, it is difficult to define, but most members have a Y-shaped connective (Zahniser and Dietrich 2013).

In recent years, many new species of the Athysanini tribe have been discovered in China, for example, *Bambusananus bispinosus* Xing & Li, *Nakaharanus lii* Wei & Xing, *Watanabella curvatua* Zhang & Xing, *Fuscmacula biprocessa* Yao, Zhang & Xing (Xing and Li 2016; Wei and Xing 2019; Zhang and Xing 2020; Yao et al. 2021). Currently, there are 29 genera and 106 species in China (Huang et al. 2025). A study of samples collected from Xizang Autonomous Region and Sichuan Province, China revealed the presence of a new genus and two new species, which are described in the present paper. The new genus is similar externally to *Nakaharanus* Ishihara but differs in characters of the male genitalia.

## ﻿Material and methods

Dry male and female specimens were used for the descriptions and illustrations. External morphology was observed under a stereoscopic microscope, and characters were measured with an ocular micrometer. The genital segments of the examined specimens were macerated in 10% NaOH. Color pictures of the adult habitus and the genitalia of specimens were obtained using a KEYENCE VHX-6000 system. Illustrations were imported into Adobe Photoshop CS8 for labeling and plate composition. The type specimens of the new species are deposited in the Institute of Entomology, Guizhou University, Guiyang, China (GUGC). A key to species is provided.

The morphological terminology used in the descriptions mainly follows Li et al. (2011). Absolute measurements, in millimeters (mm), are reported for the body.

## ﻿Taxonomy

**Tribe Athysanini Van Duzee, 1892**

### ﻿ Neonakaharanus gen. nov.

Taxon classificationAnimaliaHemipteraCicadellidae

2AA0A426-5972-5E7F-938F-BB33A6A4CBA1

https://zoobank.org/8F468EC2-E07A-4BD9-9345-106FA16CFD38

#### Type species:

*Neonakaharanus curvatus* sp. nov.

#### Description.

Colour generally yellowish brown with darker brown markings, paler in female. Postclypeus pale to blackish brown with small yellow-brown patches. Pronotum posteriorly darker. Forewing brownish hyaline with irregular darker brown markings and paler hyaline patches, markings include a dark brown patch slightly distad of midlength on costal margin; veins brown.

***Body stout*.** Length (including tegmen): ♂, 4.29–4.39 mm; ♀, 4.42–4.91 mm.

***Head*** including eyes similar in width to pronotum; crown slightly triangularly produced, shagreen, ocelli on anterior margin, situated approximately 2x their own diameter from corresponding eye; face slightly wider than long; anteclypeus slightly expanded from base to subapex. Pronotum with anterior margin strongly roundly produced, posterior margin very slightly concave, shagreen. Scutellum wider than long, its length slightly shorter than medial length of pronotum, transverse suture distinct. Forewings with four apical cells, other cells obscured by reticulate venation. Profemur with 2 dorsoapical setae, row AM with 1 stout seta, intercalary row with 13 setae, and row AV with several short setae in basal half. Hind femur moderately flattened and slightly bowed, apical setal formula 2+2+1. Hind tibia nearly straight, row PD with 12 long macrosetae and 0–3 shorter stout setae between each long seta; row AD with approximately 15 long stout setae and 0–2 shorter stout setae between each long seta.

***Male genitalia*.** Male pygofer relatively short, side slightly longer than wide, with numerous macrosetae, ventral margin concave medially. Valve subtriangular. Subgenital plate elongate, triangular, gradually tapered to narrowly rounded apex. Connective Y-shaped with stem short, sinuate in lateral view. Style relatively narrow throughout length, preapical lobe weakly developed, apophysis curved laterally, tapered to apex, hook-like. Aedeagus with shaft short, curved dorsally with a pair of apical or subapical processes and a process medially on dorsal surface, gonopore apical; dorsal apodeme weak to moderately well developed.

***Female genitalia*.** Female pygofer with ventroposterior margin slightly incurved. First valvula sculpture strigate. Second valvulae blade-like, teeth prominent extending over about distal half; dorsal sclerotized and hyaline area present. Third valvula with expanded region extending over distal half.

#### Remarks.

The new genus is indistinguishable from *Nakaharanus* in general appearance, e.g., both being robust with extra forewing cross veins, but the new genus differs in the male genitalia having the posteroventral margin of the male pygofer side without a process (present in *Nakaharanus*) and the aedeagal shaft shorter and broader with processes subapical rather than apical as in *Nakaharanus* (see Wei and Xing 2019).

#### Etymology.

The genus name refers to the similarity of the new genus to the genus *Nakaharanus*.

#### Distribution.

Palaearctic region (China).

### ﻿Key to species of *Neonakaharanus*

**Table:** 

1	Aedeagal shaft with subapical processes short (Fig. 29); dorsal surface with a medial triangular sheet-like process at midlength (Fig. 30). Female seventh sternite with posterior margin slightly sinuate (Fig. 36)	***P. triangulatus* sp. nov.**
–	Aedeagal shaft with subapical processes very long (Fig. 9); dorsal surface without a medial process. Female seventh sternite with posterior margin triangularly produced laterally (Fig. 16)	***P. curvatus* sp. nov.**

### ﻿ Neonakaharanus curvatus sp. nov.

Taxon classificationAnimaliaHemipteraCicadellidae

D93CC8BC-65A2-526F-B747-CBE22D9ACCEE

https://zoobank.org/888C9ADF-F7CE-428B-8405-A241F1101F27

Figs 1–10, 11–20, 21

#### Description.

Colour and external characters as in generic description.

***Male genitalia*** as in generic description with aedeagal shaft distally digitate, with pair of elongate subapical processes, sharply curved dorsally and converging apically, robust basally and strongly tapered distally to sharply pointed apex, shaft without process on dorsal surface (Figs 7, 9).

***Female genitalia*** as in generic description with seventh sternite tapered distally with posterior margin triangularly produced laterally (Fig. 16).

***Measurement (mm)*.** Length (including tegmen): ♂, 4.39; ♀, 4.67–4.91.

#### Type material.

**China**: • 1♂ ***(holotype)***, Xizang Autonomous Region, Linzhi City, Bayi; 10 August 2017, coll. Zaihua Yang (GUGC). ***Paratypes***: • 4♀♀, same data as holotype; (GUGC); • 1♂, Xizang Autonomous Region, Linzhi City, Bayi; 3 July 2012, coll. Maofa Yang (GUGC).

#### Distribution.

China: Xizang.

#### Remarks.

This new species can be distinguished from the male of the latter species by the aedeagal shaft without a process on the dorsal surface and the apex of the aedeagal shaft with a pair of long dorsally curved processes.

#### Etymology.

The species name is derived from the Latin word “*curvatus*”, referring to the strongly curved aedeagal processes.

### ﻿ Neonakaharanus triangulatus sp. nov.

Taxon classificationAnimaliaHemipteraCicadellidae

38615CB4-E960-5A7C-9C35-7D7EBAF8975D

https://zoobank.org/6A9BBCBF-F0E2-488E-AC76-F3494DC89971

Figs 22–30, 31–40

#### Description.

Colour and external characters as in generic description.

**Figures 1–10.**
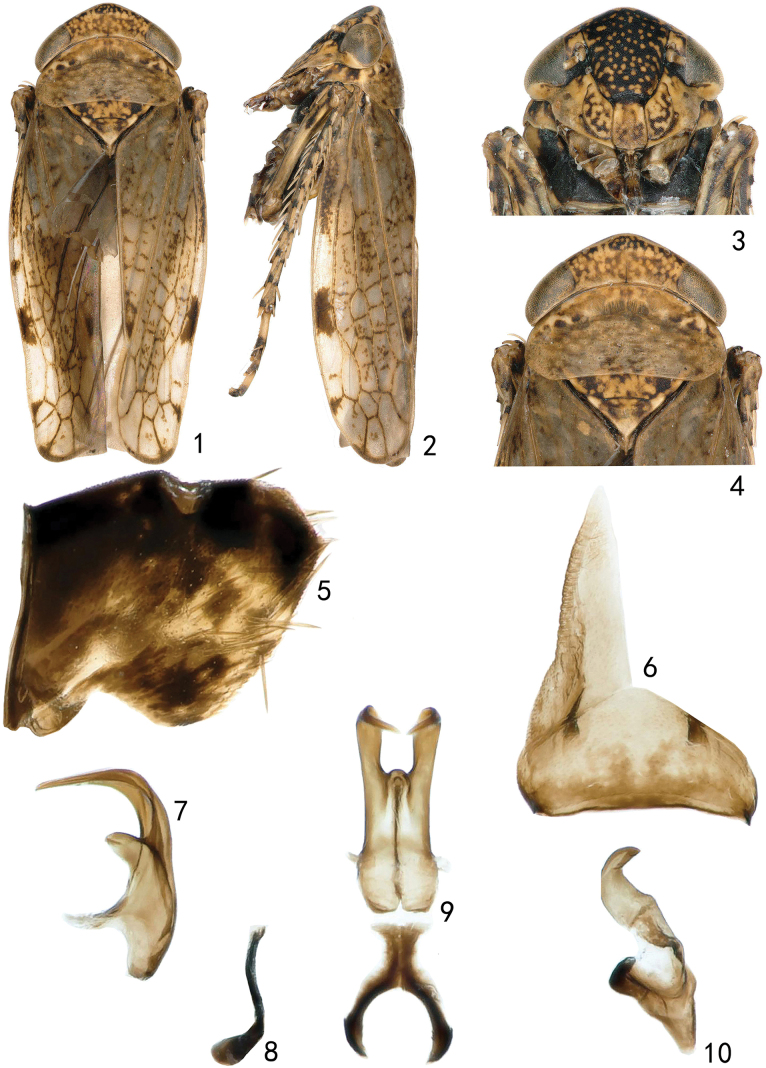
*Neonakaharanus curvatus* sp. nov. (male). **1.** Habitus, dorsal view; **2.** Habitus, lateral view; **3.** Face, ventral view; **4.** Head and thorax, dorsal view; **5.** Pygofer side, lateral view; **6.** Valve and subgenital plate, ventral view; **7.** Aedeagus, lateral view; **8.** Connective, lateral view; **9.** Aedeagus and connective, dorsal view; **10.** Style, dorsal view.

**Figures 11–20.**
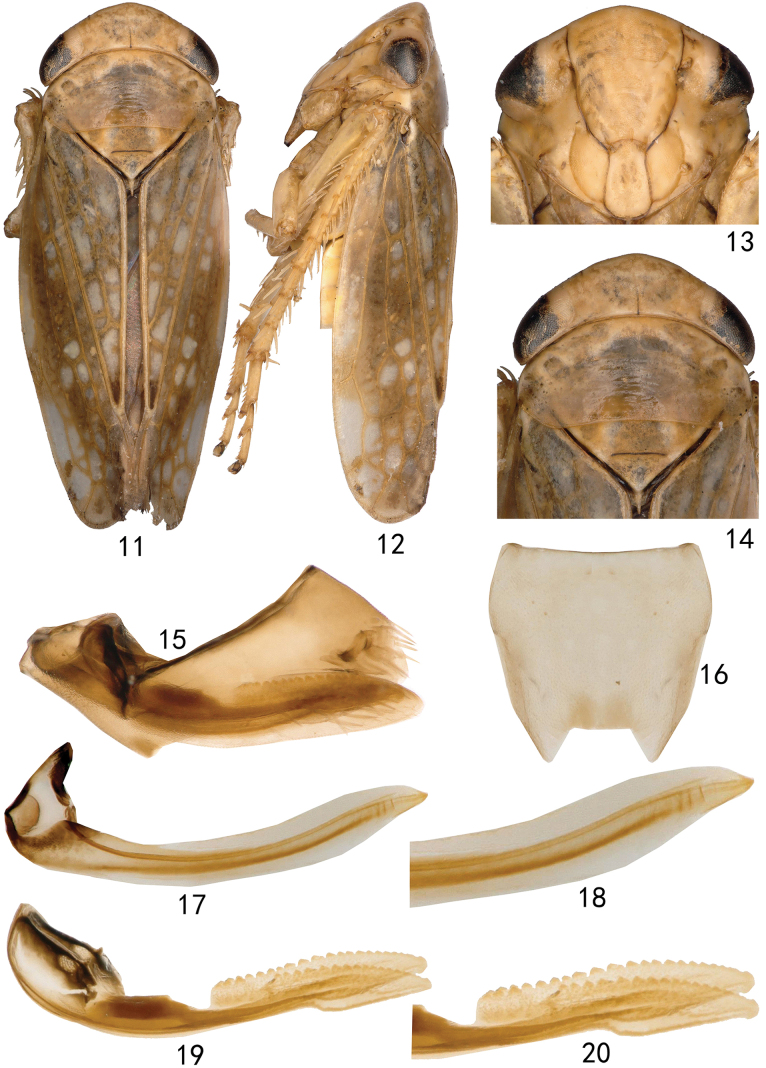
*Neonakaharanus curvatus* sp. nov. (female). **11.** Habitus, dorsal view; **12.** Habitus, lateral view; **13.** Face, ventral view; **14.** Head and thorax, dorsal view; **15.** Genital capsule, lateral view; **16.** Seventh sternite, ventral view; **17.** First valvula, lateral view; **18.** Detail of sculpture of first valvula; **19.** Second valvulae, lateral view; **20.** Detail of sculpture of second valvulae.

**Figures 21–30.**
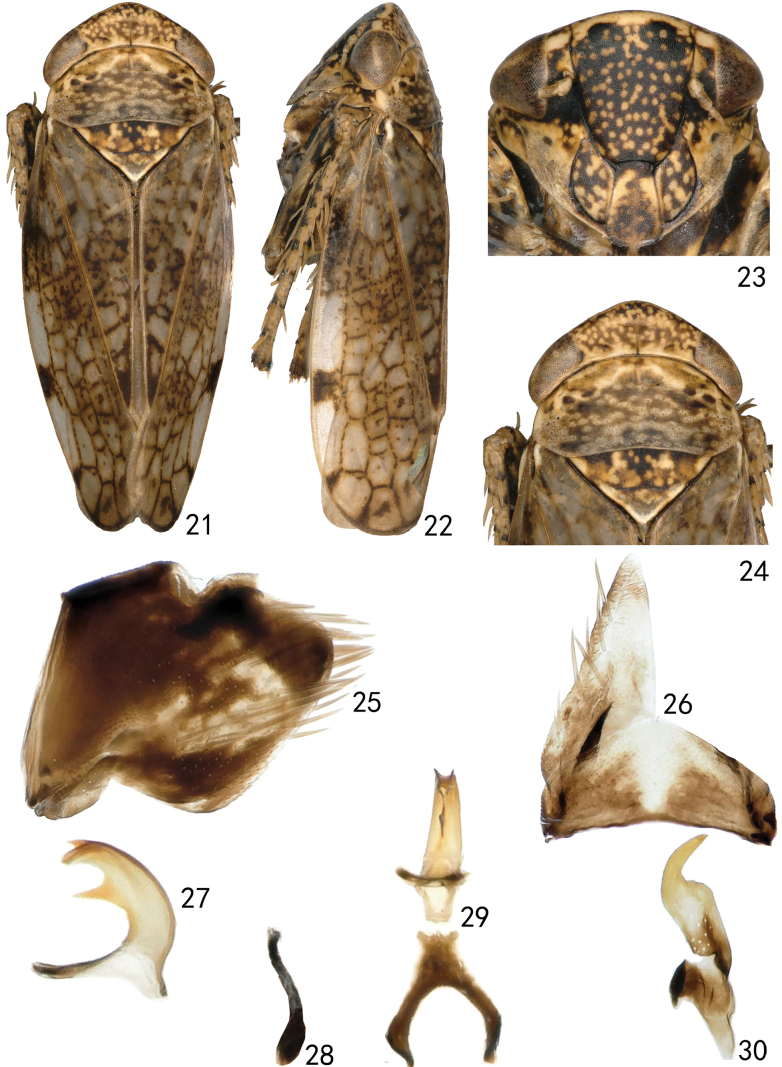
*Neonakaharanus triangulatus* sp. nov. (male). **21.** Habitus, dorsal view; **22.** Habitus, lateral view; **23.** Face, ventral view; **24.** Head and thorax, dorsal view; **25.** Pygofer side, lateral view; **26.** Valve and subgenital plate, ventral view; **27.** Aedeagus, lateral view; **28.** Connective, lateral view; **29.** Aedeagus and connective, dorsal view; **30.** Style, dorsal view.

**Figures 31–40.**
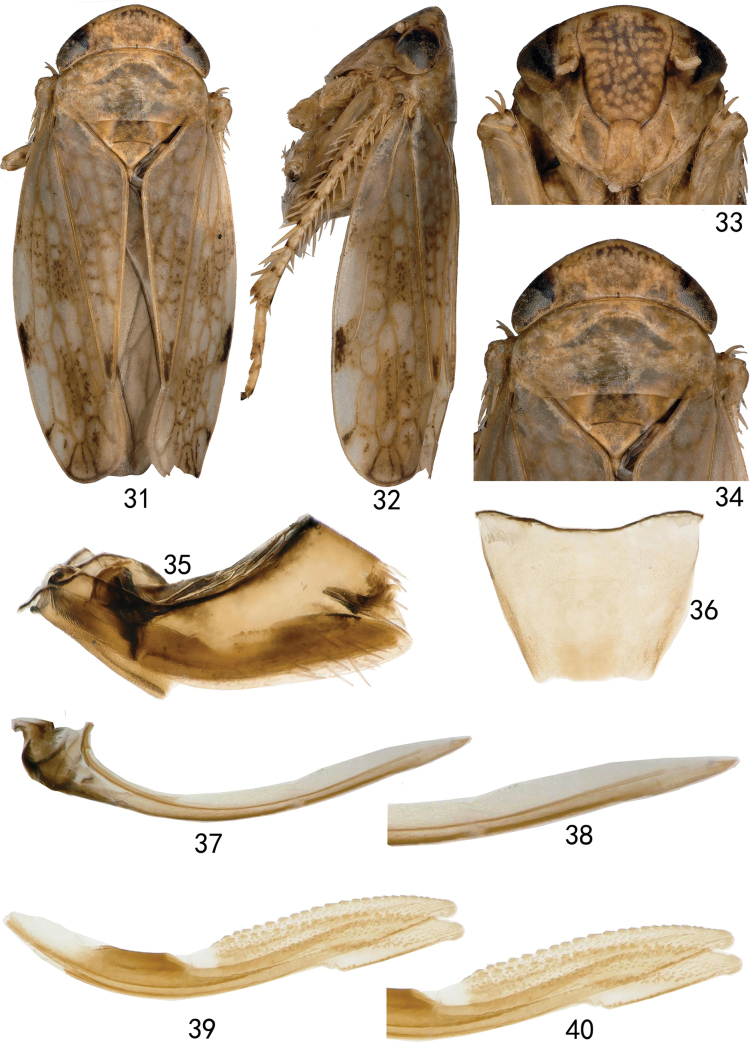
*Neonakaharanus triangulatus* sp. nov. (female). **31.** Habitus, dorsal view; **32.** Habitus, lateral view; **33.** Face, ventral view; **34.** Head and thorax, dorsal view; **35.** Genital capsule, lateral view; **36.** Seventh sternite, ventral view; **37.** First valvula, lateral view; **38.** Detail of sculpture of first valvula; **39.** Second valvulae, lateral view; **40.** Detail of sculpture of second valvulae.

***Male genitalia*** as in generic description with aedeagal shaft curved, C-shaped in lateral view, with a medial triangular sheet-like process on dorsal side and a pair of short spine-like processes subapically on ventral surface (Figs 27, 29).

***Female genitalia*** as in generic description with seventh sternite tapered distally with posterior margin slightly sinuate (Fig. 36).

***Measurement (mm)*.** Length (including tegmen): ♂, 4.29–4.37; ♀, 4.42–4.87.

#### Type material.

**China**: • 1♂ (***holotype***), Sichuan Prov., Mianyang City, Pingwu; 25 July 2016, coll. Hongping Zhan (GUGC). ***Paratypes***: • 1♂3♀♀, same data as holotype (GUGC); • 1♂, Sichuan Prov., Kangding City; 30 July 2012, coll. Zhihua Fan (GUGC); • 1♂4♀♀, Sichuan Prov., Ya’an City, Tangjia; 11 August 2015, coll. Hongping Zhan (GUGC).

#### Distribution.

China: Sichuan.

#### Remarks.

This new species can be distinguished from the male of the former species by the aedeagal shaft with a triangular sheet-like process medially on the dorsal side and the apex of the aedeagal shaft with a pair of short processes.

#### Etymology.

The species name is derived from the Latin word “*triangulatus*”, referring to the aedeagal shaft with a triangular sheet-like process on the dorsal side.

## ﻿Discussion

The new genus, along with *Nakaharanus*, can be distinguished from other Phlepsiini (in Zahniser and Dietrich 2013) by the following characteristics: in the new genus, the anterior margin of the head is smooth, whereas in Phlepsiini it is transversely striate or carinate; the head is similar in width to the pronotum, while in Phlepsiini it is distinctly narrower; and the antennal ledge is smooth, but carinate in Phlepsiini. Based on the above description, we agree with the opinion of Zahniser and Dietrich (2013) and place the new genus in Athysanini.

## Supplementary Material

XML Treatment for Neonakaharanus

XML Treatment for Neonakaharanus curvatus

XML Treatment for Neonakaharanus triangulatus

## ﻿Additional information

### Use of AI

No use of AI was reported.

### Author ORCIDs

Ke-Ting Duan https://orcid.org/0009-0008-8381-8890

Mick D. Webb https://orcid.org/0000-0002-1312-6142

Ji-Chun Xing https://orcid.org/0000-0002-3738-7996

### Data availability

All of the data that support the findings of this study are available in the main text.
